# nNOS/GSNOR interaction contributes to skeletal muscle differentiation and homeostasis

**DOI:** 10.1038/s41419-019-1584-3

**Published:** 2019-05-01

**Authors:** Costanza Montagna, Salvatore Rizza, Claudia Cirotti, Emiliano Maiani, Maurizio Muscaritoli, Antonio Musarò, Maria Teresa Carrí, Elisabetta Ferraro, Francesco Cecconi, Giuseppe Filomeni

**Affiliations:** 1 Cell Stress and Survival Unit, Danish Cancer Society Research Center, 2100 Copenhagen, Denmark; 2 Institute of Sports Medicine Copenhagen, Bispebjerg Hospital, 2400 Copenhagen, Denmark; 3 Department of Biology, Tor Vergata University of Rome, 00133 Rome, Italy; 4 Fondazione Santa Lucia, IRCCS, 00143 Rome, Italy; 5 Department of Translational and Precision Medicine (formerly Department of Clinical Medicine), Sapienza University of Rome, 00185 Rome, Italy; 6 DAHFMO-Unit of Histology and Medical Embryology, Sapienza University of Rome, 00161 Rome, Italy; 7 Department of Orthopaedics and Traumatology, Hospital “Maggiore della Carità”, University of Piemonte Orientale (UPO), Novara, Italy; 8 Department of Pediatric Hematology and Oncology, IRCCS Bambino Gesù Children’s Hospital, Rome, Italy

**Keywords:** Post-translational modifications, Ageing

## Abstract

Neuronal nitric oxide synthase (nNOS) plays a crucial role in the maintenance of correct skeletal muscle function due, at least in part, to *S*-nitrosylation of specific protein targets. Similarly, we recently provided evidence for a muscular phenotype in mice lacking the denitrosylase *S*-nitrosoglutathione reductase (GSNOR). Here, we demonstrate that nNOS and GSNOR are concomitantly expressed during differentiation of C2C12. They colocalizes at the sarcolemma and co-immunoprecipitate in cells and in myofibers. We also provide evidence that GSNOR expression decreases in mouse models of muscular dystrophies and of muscle atrophy and wasting, i.e., aging and amyotrophic lateral sclerosis, suggesting a more general regulatory role of GSNOR in skeletal muscle homeostasis.

## Introduction

The role of nitric oxide (NO) in skeletal muscle homeostasis has been deeply investigated, and findings of the last decades support the hypothesis that it is involved in both muscle contraction and atrophy^1–4^. In rat models of denervation- and disuse-induced atrophy^5^, and in dystrophin-null (*mdx*) mouse models of genetic dystrophy^2,6,7^, it has been observed that the skeletal muscle specific form of neuronal NO synthase (nNOS) dislocates from the dystrophin glycoprotein complex (DGC) located at the sarcolemma^8^. This leads first to the loss of NO beneficial effects, mostly exerted via cGMP signaling (e.g., vasodilation and satellite cell proliferation)^1,9^, and second to nitration and hyper-*S*-nitrosylation of several proteins, including those involved in Ca^2+^ release (i.e., type 1 ryanodin receptor, RyR1)^10^, in stress response and apoptosis (e.g. NF-κB and FoxO3)^5,11^.

We previously demonstrated that mice lacking the denitrosylase *S*-nitrosoglutathione reductase (GSNOR) show muscular atrophy characterized by atrogenes expression, mitochondrial alteration, and apoptosis^12^, suggesting that defective denitrosylation affects skeletal muscle function. Interestingly, we also observed that two mouse models of genetic dystrophies, namely the *mdx*^13^ and α-sarcoglycan-deficient (α-SG^−/−^) mice^14^, showed excessive levels of *S*-nitrosylated proteins (PSNOs) similar to those detected in GSNOR-null (*Gsnor*^−/−^) mice, suggesting that aberrant *S*-nitrosylation is a hallmark of muscle wasting.

It has been reported that GSNOR and nNOS co-localize with type 2 ryanodine receptor (RyR2) along the T-tubular invaginations of cardiac myocytes, this being crucial for regulation of vascular tone and cardiac contractility^15–17^. These results strongly suggest that GSNOR and nNOS act in concert to dynamically regulate NO flux and convey it on specific targets.

Here we provide evidence that GSNOR and nNOS co-immunoprecipitate and co-localize, reasonably at the sarcolemma, and that GSNOR expression is required for correct skeletal muscle differentiation and homeostasis.

## Results and discussions

### *S*-nitrosylation increase and GSNOR reduction are events associated with muscular atrophy and aging

**Fig. 1:**
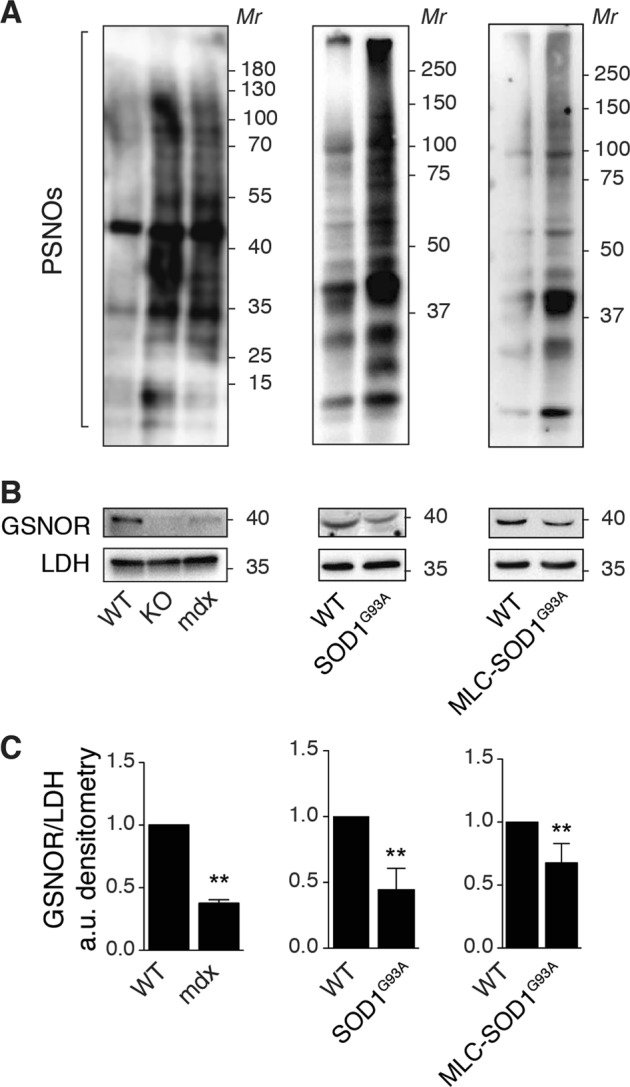
Evaluation of *S*-nitrosylation and GSNOR expression in mouse models of neuromuscular diseases. Total homogenates of gastrocnemius from 2-month-old wild-type (WT), *Gsnor*^−/−^ (KO), *mdx* mice, and mouse models of *f*ALS expressing the G93A-SOD1 mutant, either systemically (SOD1^G93A^), or exclusively in the skeletal muscle (MLC-SOD1^G93A^) were used for: **a** Biotin-switch assays of *S*-nitrosylated proteins (PSNOs) revealed, upon biotynilation, by incubation with horseradish peroxidase (HRP)-conjugated streptavidin. **b** Western blot analyses of GSNOR levels. Lactate dehydrogenase (LDH) was selected as a loading control both in (**a**) and (**b**). Results shown are representative of 3 that gave similar results. (**c**) Densitometry of GSNOR bands shown in (B), calculated by FiJi analysis software. Values shown are the means±SD of *n* = 3 different experiments normalized to LDH

We previously reported that skeletal muscles from *mdx* and αSG^−/−^ dystrophic mice show PSNOs increase that correlates with a decrease in GSNOR expression^12^. To give strength to this observation, we evaluated PSNOs and GSNOR levels in another in vivo model of progressive muscle atrophy. Particularly, we focused on mouse models of familial amyotrophic lateral sclerosis (*f*ALS) expressing the G93A-SOD1 mutant^18^, either systemically or exclusively in the skeletal muscle (MLC-SOD1^G93A^)^19^. Biotin switch assays and western blot analyses of gastrocnemius indicate that, similarly to *Gsnor*^−/−^ (KO) and *mdx* mice, both *f*ALS models show an increase of PSNOs (Fig. 1a). These results inversely correlate with GSNOR levels (Fig. 1b, c), supporting the idea that decreased GSNOR expression and the resulting excessive *S*-nitrosylation are two signatures of atrophic muscle.

**Fig. 2:**
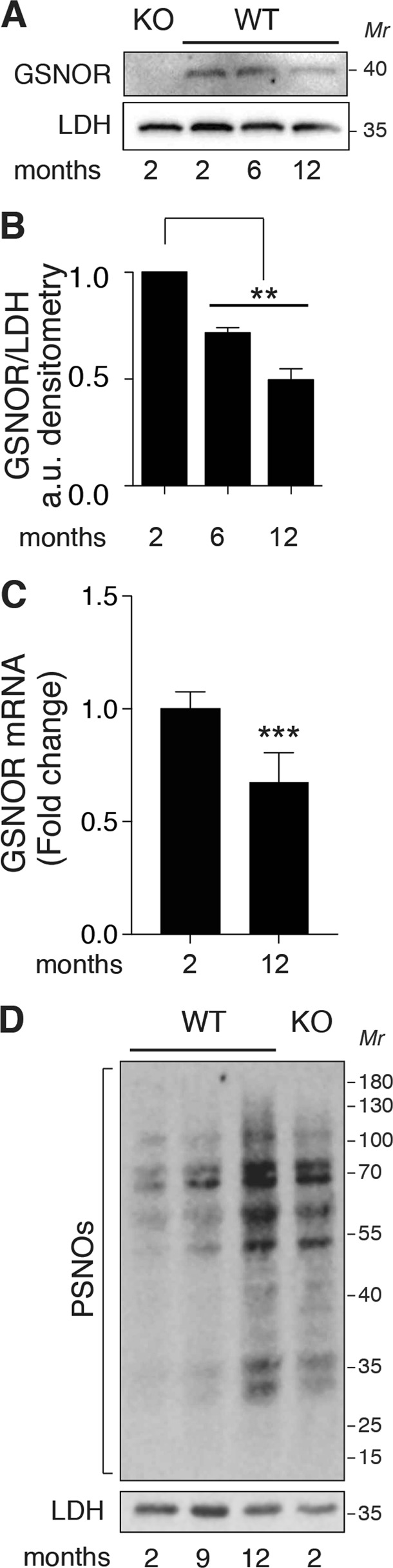
Age-dependent modulation of *S*-nitrosylation and GSNOR expression. **a** Representative western blot of GSNOR levels in gastrocnemius homogenates from 2-, 6-, and 12-months-old WT mice. Homogenates obtained from 2-months-old *Gsnor*^−/−^ (KO) muscle are shown as negative control of GSNOR expression. Lactate dehydrogenase (LDH) was selected as a loading control. **b** Densitometry of GSNOR bands shown in **a**, calculated by FiJi analysis software. Values shown are the means±SD of *n* = 3 different experiments normalized to LDH. **c** RT q-PCR analysis of GSNOR performed in gastrocnemius homogenates from 2- and 12-months-old WT mice. Results shown are the means±s.e.m. of *n* *=* 6 animals for each group. ***p *<* 0.001. **d** Representative biotin-switch assay of *S*-nitrosylated proteins (PSNOs) performed in total homogenates of gastrocnemius obtained from 2-months-old *Gsnor*^−/−^ (KO), and from 2-, 6-, and 12-months-old WT mice (WT)

Muscular atrophy is a condition usually associated with physio-pathological states related to disuse (e.g. aging), in which regeneration rate is decreased and skeletal muscle size and performance coherently reduced^20,21^. We also have recently demonstrated that GSNOR expression levels are reduced during aging. As a consequence, protein *S*-nitrosylation increases, this being a distinctive feature of aging in mammals^22^. Real-time qPCR and western blot analyses performed in skeletal muscle from 2-to-12 months old WT mice indicate that GSNOR mRNA and protein levels decrease with age also in this tissue (Fig. 2a–c). Coherently, PSNOs increase (Fig. 2d), suggesting that GSNOR hypo-expression is generally associated with a dysfunctional/aged skeletal muscle.

### GSNOR and nNOS co-localize and co-immunoprecipitate in the skeletal muscle and in myoblasts

**Fig. 3:**
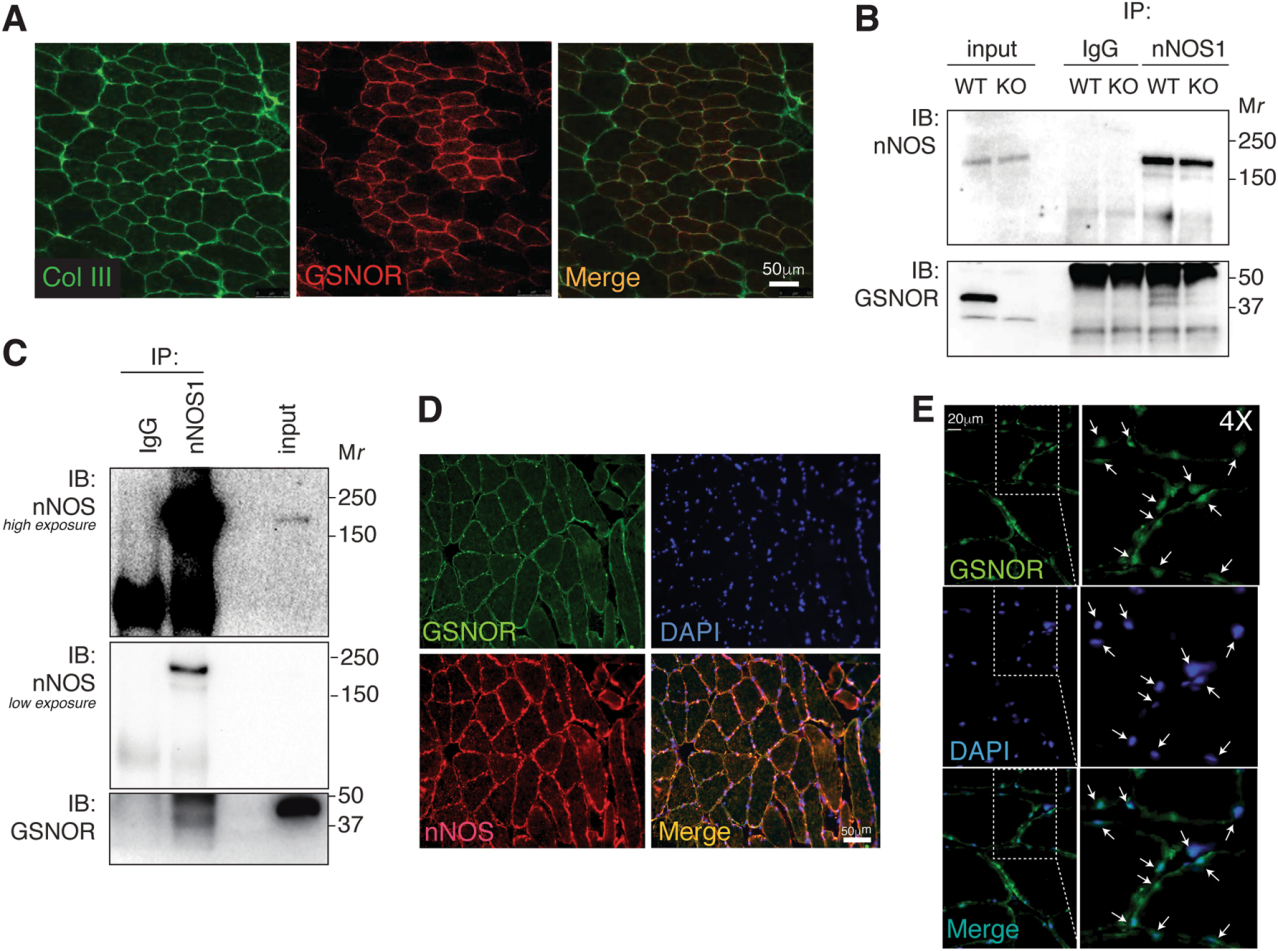
GSNOR localization in skeletal muscle. **a** Representative fluorescence microscopy images of tibialis anterior sections from wild-type (WT) mice stained with anti-GSNOR and anti-collagen III (Col III) antibodies. **b** Immunoprecipitation (IP) of tibialis anterior homogenates from 2-months-old WT and *Gsnor*^−/−^ (KO) mice performed with an anti-nNOS antibody and revealed by western blot (IB) for GSNOR and nNOS. *Gsnor*^−/−^ (KO) muscles were selected as control. **c** Immunoprecipitation (IP), as described in **b**, of lysates from C2C12 myoblasts induced to differentiate upon 4 days of serum deprivation. **d** Representative fluorescence microscopy images of human specimens of rectus abdominis stained with anti-GSNOR and anti-nNOS antibodies, or **e** with anti-GSNOR and DAPI to highlight the nuclear/perinuclear localization

*S*-nitrosylation is a posttranslational modification whose extent depends on the balance between the rates of NO production and denitrosylation, with the latter reaction largely catalyzed by GSNOR^23–25^. In the skeletal muscle, nNOS is the main enzyme responsible for NO production, which predominantly shows a sarcolemmal distribution^26^. Based on previous results suggesting an interaction between GSNOR and nNOS in cardiomyocytes^16,27^, we hypothesized that, even in the skeletal muscle, GSNOR regulates *S*-nitrosylation extent by positioning in close contact with nNOS. Therefore, we first investigated the localization of GSNOR. Immunofluorescence analyses of tibialis anterior sections show that GSNOR localizes at the sarcolemma (Fig. 3a), where also Collagen III is located. We previously showed that GSNOR deficiency did not produce any alterations in sarcolemmal nNOS distribution^12^. This suggests that, notwithstanding the same localization, GSNOR does not apparently affect nNOS attachment to DGC and, in turn, subcellular localization of NO production.

On the basis of this result, we performed co-immunoprecipitations in tibialis anterior lysates and in differentiating C2C12 mouse myoblasts. Results shown in Fig. 3b, c indicate, for the first time, that the two proteins were present in the same complex, suggesting that GSNOR – although not affecting nNOS localization – may still modulate NO effects on proteins located at the DGC. Interestingly, we were not able to observe any co-immunoprecptation in C2C12 cells before 4 days of differentiation, time at which the process of myogenesis is in an advanced state. This suggests that the presence of GSNOR and nNOS in the same complex might play a role in the late phases of differentiation. In support to these results, immunofluorescence analyses of human specimens of rectus abdominis confirmed that GSNOR co-localizes with nNOS (Fig. 3d) and, in line with previous reports^28^, also shows a (peri)nuclear distribution (Fig. 3e).

### GSNOR contributes to muscle cell differentiation

**Fig. 4:**
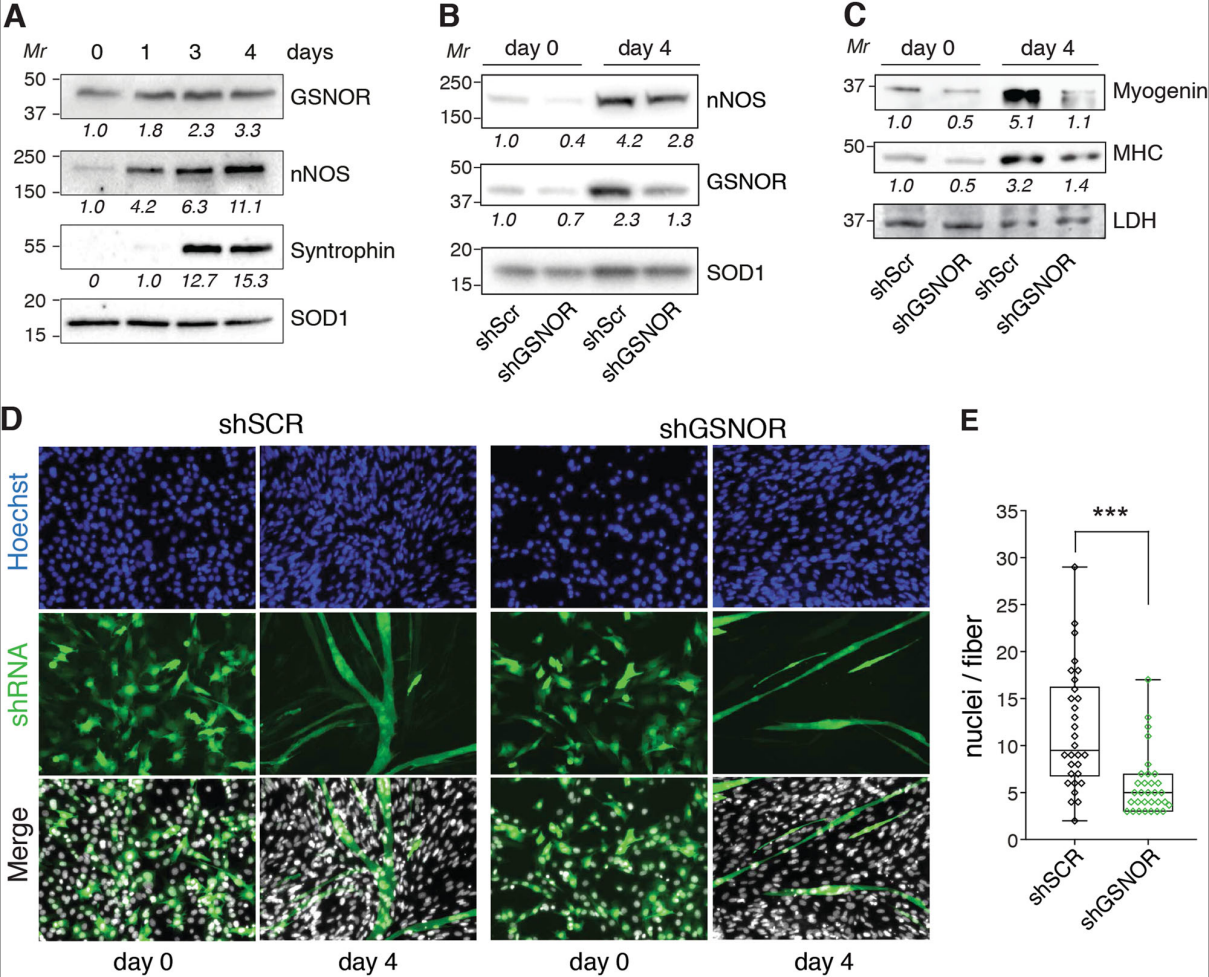
GSNOR effects on C2C12 differentiation. **a** Representative western blot analysis of GSNOR and nNOS in cell lysates from C2C12 upon serum deprivation-induced differentiation. Undifferentiated myoblasts (day 0); differentiating myotubes (day 1–4). Syntrophin and superoxide dismutase 1 (SOD1) were selected as differentiation and loading control, respectively. **b**, **c** Representative western blot analysis of nNOS, GSNOR (**b**) and myogenin and myosin heavy chain (MHC) (**c**) in non-differentiated shGSNOR and shScr C2C12 (day 0), or in the same cells after 4-days of serum deprivation-induced differentiation (day 4). SOD1 and LDH were selected as a loading control. Densitometry of each lane intensity is shown beneath the western blots. It was calculated by FiJi analysis software, normalized to LDH and arbitrarily set to 1.0 in shScr cells. **d** Immunofluorescence analysis of shGSNOR and shScr myoblasts (day 0), or 4-days differentiated myotubes (day 4). Nuclei were visualized upon staining with Hoechst 33342. Transfected cells were visualized taking advantage of the green-fluorescence of shRNAs. Superimposition (Merge) was used to calculate the fusion index, shown on the right. **e** Quantification of the data. Results are expressed as number of nuclei per fiber and represent the means±s.d. of *n* *=* 30 fibers (myotubes). ***p *<* 0.001

To verify the hypothesis that GSNOR is directly involved in skeletal muscle differentiation and homeostasis, we investigated about the existence of an integrated regulation between GSNOR and nNOS. To this end, we evaluated their levels by western blot analysis in differentiating C2C12 mouse myoblasts. Figure 4a shows that nNOS and GSNOR increase time-dependently in parallel with syntrophin (used as marker of differentiation). Next, we downregulated GSNOR by short-hairpin RNA (shRNA) (Fig. 4b), and analyzed if this induced any alterations in C2C12 differentiation. Western blot analyses of myogenin and myosin heavy chain (MHC) – two proteins required respectively for commitment and differentiation of myogenic precursor cells^29^ – indicate that their expression is decreased upon GSNOR knocking-down (Fig. 4c). Of note, GSNOR-depleted (shGSNOR) C2C12 cells display a reduction in nNOS levels (Fig. 4d), exhibit a decreased number of myotubes and a lower fusion index with respect to the control (shScr) counterparts (Fig. 4e).

These results strongly argue for GSNOR playing a role in myogenesis, this being in perfect agreement with recent data^30^ and previous evidence showing that *Gsnor*^−/−^ mice show a delayed muscle regeneration following injury^12^.

## Conclusion

The role of NO and *S*-nitrosylation in skeletal muscle homeostasis has been exhaustively studied. However, the implication of GSNOR and denitrosylation remains controversial^12,31^. Here, we provide evidence that, in the same way of kinases and phosphatases (and/or ligases and de-ubiquitinylases), the denitrosylating enzyme GSNOR co-localizes with the source of NO, nNOS, to precisely control *S*-nitrosylation. Together with recent data indicating that, at least in *E.coli*, *S*-nitrosylation is an enzymatically driven process^32^, our results represents a further evidence that *S*-nitrosylation is a finely controlled posttranslational modification which is required, in the skeletal muscle, to maintain correct myofiber function and homeostasis. Such a fine regulation is lost when nNOS dislocates from sarcolemma, or – as here reported – when GSNOR is downregulated. Actually, besides excessive *S*-nitrosylation, other NO-mediated mechanisms concur to muscular atrophy, which we cannot exclude might also play a role in GSNOR-deficient systems, such as: (1) deactivation of cGMP signaling, which is extremely important to sustain vasodilation^1,26^ and stimulate satellite cells proliferation^9^; (2) deregulation of Ca^2+^ uptake/release for sarcoplasmic reticulum^1,33^; (3) impairment of mitochondrial biogenesis and metabolism^34^.

**Fig. 5:**
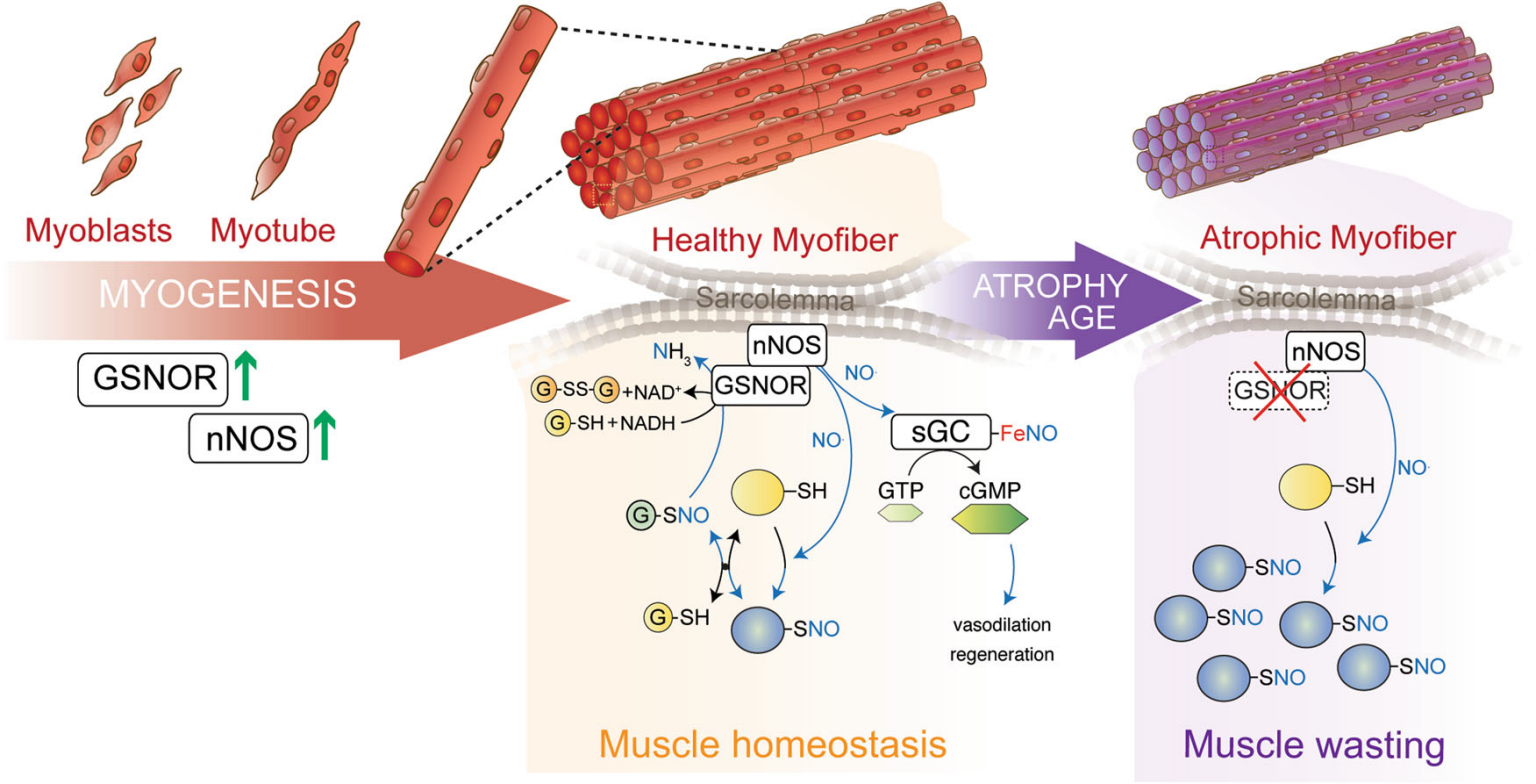
Schematic model of GSNOR function in skeletal muscle differentiation and homeostasis. Our data indicate that GSNOR and nNOS are co-expressed and reasonably part of the same complex (co-immunoprecipitate) during myogenesis. In this way, they sustain differentiation (left). Co-immunoprecipitation and co-localization analyses indicate that GSNOR and nNOS might be recruited in the same complex close to the sarcolemma. This, along with the correct activation of NO/cGMP signaling induced by sarcolemmatic nNOS, should allow the nitrosylation of specific targets (e.g. RyR1) and contribute to the correct physiology of the skeletal muscle (e.g., vasodilation and regeberation) (middle). During aging, or in dystrophic muscles, GSNOR levels are found to be reduced and, coherently, the extent of *S*-nitrosylated protein (PSNO) increased (right). GSNOR decrease is a hallmark of patho-physiological states associated with muscle wasting. However, we still do not know if this event is associated with nNOS translocation into the sarcoplasm, as reported in different pathological models of atrophy. Although reasonable, so far there is no evidence supporting this hypothesis

In line with data arguing for a pivotal role of GSNOR and *S*-nitrosylation in myoblast differentiation^30^ and muscle regeneration^12,31^, we also observed that GSNOR is involved in myogenesis. This is probably due to its recruitment in the same complex with nNOS, which becomes detectable at day 4 when other markers of differentiation are still expressed (Fig. 5). This evidence suggests that nNOS and GSNOR start to functionally interact close to the sarcolemma in the late phases of muscle differentiation. GSNOR is found decreased in aging and in genetic models of muscular atrophy, this allowing to speculate that this phenomenon contributes to skeletal muscle homeostasis by conveying NO signal on specific protein thiols located underneath the sarcolemma (Fig. 5). Linked to this, it has been demonstrated that mesenchymal stem cells from *Gsnor*^−/−^ mice exhibit lower adipogenic versus higher osteogenic differentiation, due to an inhibitory *S*-nitrosylation at Cys139 in PPARγ^35^. However, *Gsnor*^−/−^ mice are smaller than WT counterparts and exhibit bone loss due to an increased number of osteoclasts^36^. This suggests that selective *S*-nitrosylation underlies skeletal muscle homeostasis by means of at least two different mechanisms: (i) directly, as above mentioned, by affecting stem cell differentiation and tissue regeneration; (ii) indirectly, at systemic level, by interfering with the mechanic stimuli aimed at inducing an appropriate skeletal muscle implant into the bone.

In agreement with these observations, two-month-old *Gsnor*^−/−^ mice exhibit both osteopenia^35^ and sarcopenia^12^, which represent pathological states related to aging. We recently reported that GSNOR is physiologically silenced in mouse and human aging due to downregulation of the DNA demethylase, ten-eleven translocation protein 1 (Tet1)^22^. Remarkably, *Tet1*^−/−^ mice are smaller than WT animals, resembling, in such an aspect, *Gsnor*^−/−^ mice. This feature is associated with developmental skeletal muscle defects and deregulated expression of muscle contraction genes, which correlate with an augmented methylation status of the DNA^37^. Here, we have coherently provided evidence that in mouse models of muscular dystrophies, or physio-pathological states associated with skeletal muscle wasting (i.e. aging and ALS), GSNOR expression is decreased. These results argue for GSNOR being a molecular determinant of skeletal muscle homeostasis, with any dysregulations of its expression potentially affecting muscle healthy state.

## Materials and methods

### Animals

Mouse experiments were carried out in accordance with the European Community guidelines and with the approval of relevant National and local ethical committees. The *Gsnor*^−/−^ mouse strain was generated by Prof. Stamler^15,38^, while the C57BL/6 wild-type were purchased from Charles River. *mdx* and *α-SG*^−/−^ mice were from Jackson Laboratories. G93A-SOD1mice B6.Cg-Tg(SOD1 G93A)1Gur/J were purchased from The Jackson Laboratory and were kept on C57BL/6 J background. Mouse models of *f*ALS expressing the mutant SOD1^G93A^ selectively in skeletal muscle (MLC-SOD1^G93A^) were generated by Prof. Musarò^19^. Mice were housed in an environmentally controlled room (23 °C, 12 h light–dark cycle) and provided with food and water ad libitum.

### Detection of PSNOs

Protein *S*-nitrosylation extent was evaluated by biotin-switch assay as previously described^12,39^. In brief, muscles were homogenized in HEN buffer (25 mM HEPES, 50 mM NaCl, 0.1 mM EDTA1% NP-40, protease inhibitors, pH 7.4). Free cysteine residues were blocked with *S*-methyl methanethiosulfonate (MMTS, Sigma) and protein pellets, collected upon precipitation in cold acetone, were re-suspended in HENS buffer (HEN buffer with 1% SDS) and let react with biotin-HPDP, with or without ascorbate. Biotinylated proteins were revealed using the Amersham ECL detection system after incubation with the HRP-conjugated streptavidin (Merck).

### Immunoprecipitation assays

Immunoprecipitations were performed adding 1 μg of anti-nNOS antibody (Santa Cruz), or IgG, to 10 μl of prewashed Dynabeads^®^-protein G (Invitrogen). Five hundred microgram of whole tibialis anterior extracts, or 800 μg of C2C12 cell extracts, were incubated for 3 h with Dynabeads^®^-Ab complex and washed with 0.15 M NaCl, 10 mM HEPES pH 7.5. Immunoprecipitated proteins were detached from beads by boiling in sample buffer, separated by SDS-PAGE, transferred to nitrocellulose membranes (Amersham) and then incubated with anti-GSNOR or anti-nNOS antibody. IgG were used as negative control.

### RT q-PCR

Gastrocnemius was homogenized in TRI-Reagent (Sigma) and RNA was extracted in accordance with the manufacturer’s protocol. cDNA was generated using the GoScript Reverse Transcription System (Promega). RT q-PCR was performed using the iTAQ Universal SYBR Green Supermix (Bio-Rad) on ViiA 7 Real-Time PCR System (Thermo-Fisher Scientific) and data were analyzed using the second derivative maximum method. All reactions were run as triplicates and normalized to the internal standard ribosomal protein L34. Primers used are the following:

GSNOR FW-tcacttcatggggactagca, RV-ccgagggatcgattttagca;

L34 FW-ggtgctcagaggcactcaggatg, RV- gtgctttcccaaccttcttggtgt.

### Cell culture and transfection

C2C12 cells were grown in DMEM (Thermo-Fisher Scientific) supplemented with 10% FBS, 1000 U/mL penicillin-streptomycin at 37 °C in 5% CO_2_, or in 2% horse serum-containing DMEM to induce differentiation. When stated, 24 h after plating, C2C12 cells were transfected with short-hairpin RNAs against GSNOR designed in our laboratory and synthesized by Sigma.

Top strand:

5′-tgctgctcccactaccacactgacacgttttggccactgactgacgtgtcagtggtagtgggag-3′;

Bottom strand:

5′-cctgctcccactaccactgacacgtcagtcagtggccaaaacgtgtcagtgtggtagtgggagc-3′. The oligonucleotides were cloned in the pcDNA6.2-GW/EmGFP-miR vector (Thermo-Fisher Scientific) using the BLOCK-iT™ Pol II miR RNAi Expression Vector Kit with EmGFP (Thermo-Fisher Scientific) in according to manufacturer’s instructions.

### Immunofluorescence

Human tissues: muscle biopsy specimens were derived from a previous study (10.1038/srep30340). They were obtained from the M.G. Vannini Hospital in Rome (Italy), from patients who signed an informed consent, after clearance by the local ethical committee. Biopsy specimens were obtained during the initial phase of the operation from the rectus abdominis muscle of patients undergoing abdominal surgery for non-neoplastic reasons, and used as controls in the previous study. Reasons for abdominal surgery in controls were incisional hernia, cholelithiasis, benign prostatic hyperplasia, epigastric hernia and mesenteric cyst. Biopsy specimens were immediately frozen in liquid nitrogen and stored at –80 °C until analysis. Mouse tissues: tibialis anterior and gastrocnemius were embedded in O.C.T. (Bio-Optica) and flash-frozen in liquid nitrogen-cooled isopentane (VWR). All sections were cut to a thickness of 8 μm using a Leika cryostat; fixed in 4% paraformaldehyde; permeabilized in 0.2% Triton X-100/1% BSA (Sigma); blocked in 10% horse serum (Sigma); and incubated for 1 h with: anti-GSNOR (Sigma, 1:100) and anti-Collagen III (Sigma, 1:100). Afterwards, cryosections were incubated with labeled secondary antibodies (Thermo-Fisher Scientific) and examined by a Leica TCS-SP5 confocal microscopy. Fluorescence images were adjusted for brightness, contrast, and color balance using Fiji^40^. Cells: undifferentiated myoblasts (day 0) and differentiated myotubes (day 4) were fixed in 4% paraformaldehyde, stained with Hoechst 33342 (to visualize nuclei) and analyzed using an EVOS Floid Cell Imaging Station (Thermo-Fisher Scientific). Only cells/fibers expressing the GFP-tagged shRNAs were considered to quantify the fusion index, which has been evaluated by counting the number of nuclei in each fiber with Fiji analysis software^40^.

### Western blotting

Samples from gastrocnemius were homogenized, and C2C12 cells were lysed in lysis buffer containing 0.15 M NaCl, 10 mM HEPES (pH 7.5). Antibodies used: anti-nNOS, anti-SOD1, anti-LDH, anti-α-syntrophyn, anti-myogenin, anti-MHC (Santa Cruz), and anti-GSNOR (Millipore). Immune-reactive bands were revealed by Chemidoc System (Bio-Rad) and quantified by densitometry using Fiji^40^.

Protein concentration was determined by the DC™ Protein Assay (Bio-Rad).
